# COVID-19 and Its Repercussions on Oral Health: A Review

**DOI:** 10.3390/medicina57111189

**Published:** 2021-11-01

**Authors:** Laura-Cristina Rusu, Lavinia Cosmina Ardelean, Codruta Victoria Tigmeanu, Anamaria Matichescu, Iulia Sauciur, Emanuel Adrian Bratu

**Affiliations:** 1 Department of Oral Pathology, Multidisciplinary Center for Research, Evaluation, Diagnosis and Therapies in Oral Medicine, “Victor Babeș” University of Medicine and Pharmacy Timisoara, 2 Eftimie Murgu Sq., 300041 Timisoara, Romania; laura.rusu@umft.ro (L.-C.R.); sauciuriulia@yahoo.com (I.S.); 2 Department of Technology of Materials and Devices in Dental Medicine, Multidisciplinary Center for Research, Evaluation, Diagnosis and Therapies in Oral Medicine, “Victor Babeș” University of Medicine and Pharmacy Timisoara, 2 Eftimie Murgu Sq., 300041 Timisoara, Romania; tigmeanu.codruta@umft.ro; 3 Department of Preventive Dentistry, Community and Oral Health, Translational and Experimental Clinical Research Center in Oral Health, “Victor Babeș” University of Medicine and Pharmacy Timisoara, 2 Eftimie Murgu Sq., 300041 Timisoara, Romania; 4 Department of Implant Supported Restorations, “Victor Babeș” University of Medicine and Pharmacy Timisoara, 2 Eftimie Murgu Sq., 300041 Timisoara, Romania; ebratu@umft.ro

**Keywords:** COVID-19, SARS-CoV-2, pandemic, oral lesions, oral manifestations, periodontal disease, temporomandibular disorders, dental medicine

## Abstract

In 2019, a new type of coronavirus, SARS-CoV-2, the causing agent of COVID-19, was first detected in Wuhan, China. On 11 March 2020, the World Health Organization declared a pandemic. The manifestations of COVID-19 are mostly age-dependent and potentially more severe in cases with involved co-morbidities. The gravity of the symptoms depends on the clinical stage of the infection. The most common symptoms include runny nose and nasal congestion, anosmia, dysgeusia or hypogeusia, diarrhea, nausea/vomiting, respiratory distress, fatigue, ocular symptoms, diarrhea, vomiting, and abdominal pain. These systemic conditions are often accompanied by skin and mucosal lesions. Oral lesions reported in patients with COVID-19 include: herpex simplex, candidiasis, geographic tongue, aphthous-like ulcers, hemorrhagic ulcerations, necrotic ulcerations, white hairy tongue, reddish macules, erythematous surfaces, petechiae, and pustular enanthema. It is still unclear if these manifestations are a direct result of the viral infection, a consequence of systemic deterioration, or adverse reactions to treatments. Poor oral hygiene in hospitalized or quarantined COVID-19 patients should also be considered as an aggravating condition. This narrative review is focused on presenting the most relevant data from the literature regarding oral manifestations related to SARS-CoV-2, as well as the challenges faced by the dental system during this pandemic. A routine intraoral examination is recommended in COVID-19 patients, either suspected or confirmed, as, in certain cases, oral manifestations represent a sign of severe infection or even of a life-threatening condition. It is our belief that extensive knowledge of all possible manifestations, including oral lesions, in cases of COVID-19 is of great importance in the present uncertain context, including new, currently emerging viral variants with unknown future impact.

## 1. The Essentials about CoVs

Coronaviruses (CoVs) are members of the Coronaviridae family. These enveloped viruses possess a non-segmented, single-stranded, positive-sense RNA, with a unique replication strategy [1].

CoVs are known to affect different animal species and cause mild to severe respiratory infections in humans. In 2002 and 2012, two highly pathogenic coronaviruses of zoonotic origin, causing the severe acute respiratory coronavirus syndrome (SARS-CoV-1) and the Middle East respiratory coronavirus syndrome (MERS-CoV), respectively, affected humans, resulting in fatal respiratory diseases [2,3], and turning coronaviruses into a 21st century public health problem. The virus has been identified in various non-human hosts [4,5,6,7]. Extremely pathogenic CoVs belong to the genus Beta-coronavirus, group 2, which causes severe disease [8].

In 2019, a new type of coronavirus, SARS-CoV-2, a Beta-coronavirus causing the COVID-19 disease, was first detected in Wuhan, China [9]. SARS-CoV-2 is composed of 16 non-structural proteins with specific roles in replication [10]. COVID-19 has spread rapidly around the world and, on 11 March 2020, the World Health Organization declared it a pandemic [11].

The SARS-CoV-2 genome sequence shares ~80% sequence identity with SARS-CoV-1 and ~50% with MERS-CoV [12]. The structural spike protein (S), which mediates SARS-CoV’s entry into host cells, is highly variable in the case of SARS-CoV-2. Its receptor-binding domain enables direct contact with the cell receptor angiotensin-converting enzyme II (ACE2) [5,13].

ACE2, and thus any cells that express ACE2, may be target cells and therefore susceptible to COVID-19 infection [12]. Zou et al. [14] explored the expression of the ACE2 receptor on different cells from human body tissues and classified the infectious risk potential. Lung, heart, esophagus, kidney, bladder, and ileum have been considered organs at risk [14]. A high ACE2 expression was found in the oral mucosa and the epithelial cells of the tongue [15]. After entering the cell, the virus delays the immune system response, allowing the infection to progress, and it becomes much harder to fight [16].

## 2. Main Characteristics of COVID-19

### 2.1. General Characteristics

The host’s response represents an important factor in the disease process and tissue damage. In most cases of SARS-CoV-2 infection, the primary immune response leads to viral elimination. In certain patients, the secondary immune response may be exaggerated and lead to inflammation-induced lung damage, pneumonia, acute respiratory distress syndrome, respiratory failure, shock, organ failure, and possible death [17].

Severe COVID-19 is also characterized by hypercoagulopathy and neurological and/or gastrointestinal tract damage, the fatal outcome in severe cases being due to the macrophage activation syndrome, which causes a “cytokine storm” [18].

Age, acute cardiac injury, heart failure, skeletal muscle injury, and lymphopenia have been associated with mortality in COVID-19 cases [19,20].

**Figure 1:**
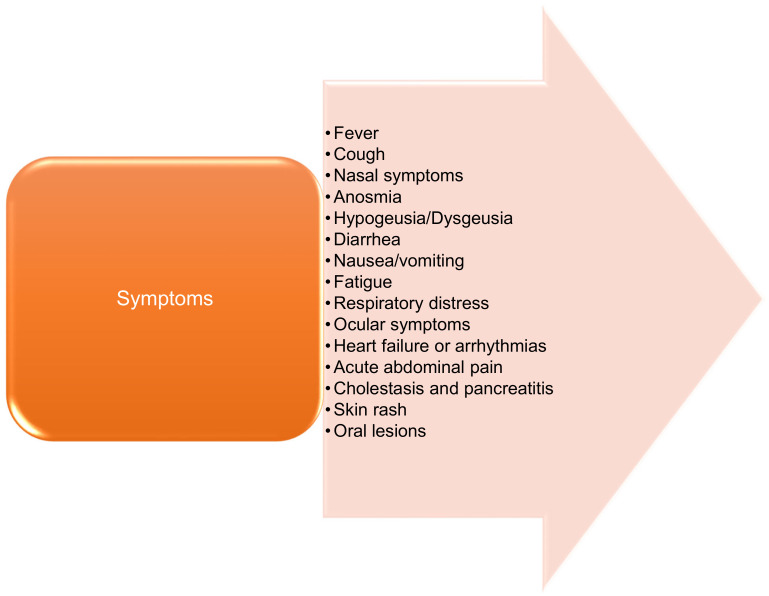
Main symptoms of COVID-19.

The most common symptoms (Figure 1) include fever, cough, runny nose and nasal congestion, anosmia, dysgeusia/hypogeusia, diarrhea, and nausea/vomiting [21,22]. Other clinical manifestations include: fatigue, ocular symptoms (conjunctival secretion), and arrhythmias. Gastrointestinal symptoms or abdominal pain can occur in the absence of respiratory symptoms. Acute cholestasis and pancreatitis have also been reported in children and adolescents [23,24].

The gravity of the symptoms depends on the clinical stage of the infection [25,26]. Moderate infection has been reported as pneumonia, without obvious hypoxemia or difficulty in breathing. Clinical signs and symptoms, as well as the thoracic CT, suggest subclinical lung lesions. In cases of severe infection, oxygen saturation is less than 92%, with manifestations of hypoxia [24]. Critical infection is characterized by respiratory failure, shock, encephalopathy, myocardial injury or heart failure, coagulation dysfunction and acute kidney damage, and multiple organ dysfunction [23].

### 2.2. Severity by Age

The manifestations of COVID-19 are mostly age-dependent and potentially more severe in cases with involved co-morbidities. The severity of the disease was found to correlate with increasing age [27]. Patients over 65 years of age have a high risk for COVID-19 infection, develop more severe forms, and show increased mortality [19,28,29] due to low immune response [30]. In addition, other factors, such as underlying cardio-vascular disease (CVD), may negatively influence the clinical outcome and explain the higher mortality rate, as CVD prevalence increases with age [20]. Older patients show higher incidences of skeletal muscle injury and acute kidney injury [19].

In the early stages of the pandemic, young patients had better clinical outcomes compared to adults, and death rates were lower. In most cases, children diagnosed with COVID-19 developed asymptomatic, mild, or moderate illnesses and recovered one or two weeks after the onset of the disease [23], the clinical symptoms being milder then in adults [26]. Children, even if asymptomatic, can transmit the disease very easily, nasal and fecal secretions being a further challenge for infection control [31].

No differences were identified between young patients and elderly patients in terms of the degree of lung damage [32] but, in elderly patients, the involvement of multiple lobes was higher [29].

As older age groups are vaccinated, children and unvaccinated populations are becoming at higher risk of contracting COVID-19, and developing severe illness. Furthermore, the more contagious Delta strain, which is dominant at present, seems to be impacting younger age groups more than previous variants, despite the fact that does not specifically target children [33].

### 2.3. Skin Involvement

The systemic conditions may be accompanied by skin lesions in which the innate immune system is involved [34,35]. Associated immune-mediated skin diseases, such as psoriasis, atopic dermatitis, and suppurative hidradenitis, have been reported [36,37].

The skin lesions most often associated with COVID-19 are morbilliform, pernio-like, urticarial, macular erythematous, vesicular, and papulosquamous lesions, as well as retiform purpura and chilblains [38,39,40,41,42,43,44,45]. Skin lesions such as maculopapular lesions and urticarial and vesicular eruptions, as well as transient livedo reticularis and acral peeling, are also frequently mentioned. In children and young adults, red-purple nodules have also been described on distal figures (sometimes called “COVID toes”), similar in appearance to perblio (chilblains) [46].

Skin manifestations are often accompanied by mucosal damage. Extensive skin lesions over the fleshy portion of the buttocks and on the mucosa of the nostrils, tongue, lips, and urethra were reported in hospitalized COVID-19 patients, despite the minimal exposure to pressure. Their extent suggests an inflammatory vascular process beyond pressure-related skin damage [47].

## 3. Oral Lesions Related to COVID-19

This narrative review is focused on presenting the most relevant data from the literature regarding oral manifestations related to SARS-CoV-2, as well as the challenges faced by the dental system during this pandemic.

An electronic search was conducted in PubMed, Scopus, and Web of Science for studies published up to April 2021, with the following keywords used: “oral lesions”, “mucosal lesions”, “COVID-19”, OR “SARS-CoV-2”. The full-text articles were evaluated, and the references cited by the relevant studies were further manually searched. Given the available data limited, only literature reviews were excluded. Articles for which the full text was not accessible or not available in English were also excluded.

Reports of different oral manifestations related to COVID-19 cases were described in the literature. Oral lesions reported in patients with COVID-19 were quite heterogeneous (Table 1), varying in the kind of lesion and location. In most cases, the pathogenesis of these manifestations was not clearly defined, being categorized as a direct result of the viral infection, a consequence of immune misbalance, or an adverse reaction to treatment [47,48,49]. Poor oral hygiene in hospitalized or quarantined COVID-19 patients should also be considered as an aggravating condition [50].

Amorim dos Santos et al. [51] described the case of multiple oral lesions in a 67-year-old man diagnosed with COVID-19. After 20 days of hospitalization, a persistent white plaque on the tongue dorsum was detected and diagnosed as fungus infection. This lesion was treated with intravenous fluconazole and oral nystatin, without any result. Multiple yellowish pinpoint ulcers, diagnosed as recurrent oral herpes, were also present on the tongue dorsum. A fibroma, about 1 cm in diameter, in the lower lip, related to previous conditions, was also observed. The tongue scraping culture presented Saccharomyces cerevisiae. These conditions were accompanied by extremely viscous saliva. As treatment, antifungals were administered and chlorhexidine digluconate (0.12%) non-alcoholic mouthwashes and daily applications of 1% hydrogen peroxide were performed. After 2 weeks, the lesions showed an almost complete remission, but severe, asymptomatic geographical tongue was observed, according to the severity index [52]. Ten days after removing the patient from hospital, the geographical tongue was still present, but it was reclassified as moderate according to the severity index. [52] The authors suggested that the oral lesions, coinfections, and secondary manifestations may have been due to systemic condition of the patient.

Ansari et al. [53] presented two cases, a 75-year-old male and a 56-year-old female, involving several painful ulcers with irregular margins and varying sizes, with red and non-hemorrhagic backgrounds, located on the hard palate and anterior region of the tongue, respectively. In case 1, the lesions appeared five days after the onset of the symptoms and, in case 2, one week after hospitalization. The diagnostic was of diffuse edema with desquamation, granulation, and ulceration under the mucosa and with invasion of mononuclear and neutrophilic cells, indicating a secondary bacterial infection. The serologic tests for herpes simplex virus (HSV-1 and HSV-2) were negative. The treatment consisted of diphenhydramine, dexamethasone, tetracycline, and lidocaine, and complete recovery was obtained after approximately 7 days. The authors suggested that the oral lesions were due to COVID-19.

Bezerra et al. [54] reported a case of a 33-year-old male patient, treated with ivermectin and azithromycin for suspicion of COVID-19, who developed a painful mouth ulceration in the floor of mouth. After 10 days of topical application of corticosteroids, the lesion showed full remission. After twenty days, ageusia was still present, and two other crateriform ulcers with a necrotic background and no erythematous halo were detected in the left retromolar region and lip mucosa. The patient did not report any history of recurrent aphthous ulceration. After 7 days of topical treatment with triamcinolone acetonide and 0.12% chlorhexidine digluconate mouthwash, the oral ulcerations showed total remission.

Brandao et al. [55] presented eight cases of different oral manifestations in patients with COVID-19.

Case 1. A case of an 81-year-old male, presenting multiple painful, shallow aphthous-like ulcers with irregular margins covered with mucopurulent membrane in the mucosa of the upper and lower lips, as well as in the anterior tongue dorsum, was reported. HSV-1 was identified by saliva testing. Treatment with intravenous acyclovir three times a day for 10 days showed no clinical improvement. To relieve the pain caused by oral ulcers, photobiomodular (PBMT) therapy was performed for 10 days. The symptoms improved after 2 days and after 11 days they were completely resolved.

Case 2. A 71-year-old female presented small hemorrhagic ulcerations on the upper and lower lips. Focal areas of superficial necrosis on the anterior tongue dorsum, developed at the time of hospitalization, were also observed. Following the PCR saliva test, HSV-1 was identified. Intravenous acyclovir was administrated three times a day for 7 days, without any results. Pain was treated with PBMT and regression was observed after 10 days of light therapy, but the lip ulcers did not heal even after 15 days of therapy.

Case 3. In the case of an 83-year-old female, the oral examination revealed an ulcer of 1.5 × 1.5 cm^2^ on the right lateral edge of the tongue, accompanied by a petechiae and a superficial necrotic area in the anterior hard palate. These lesions were painful. Following the PCR test, a negative result was obtained for the HSV-1. After 5 days of PBMT treatment, the pain started to diminish, with remission after 10 days.

Case 4. The case of a 72-year-old male with small hemorrhagic ulcerations affecting the upper and lower lips and a painful necrotic ulceration on the mucosa of the lower right lip, detected a few days after hospitalization, was described. The HSV-1 was detected following the PCR test. The patient received intravenous acyclovir treatment for 7 days, but no improvement was observed. The painful oral necrotic ulcers were treated with PBMT, and regression was observed after 7 days of light therapy.

Case 5. A 32-year-old female, isolated at home with a mild form of COVID-19, showed multiple ulcers on the tip and lateral edges of the tongue, after 10 days of treatment. A teleconsultation was performed by a dentist, following which small superficial and circular lesions with a whitish center and surrounded by an erythematous halo were observed. The patient had no history of recurrent oral ulcers, oral inflammatory diseases, or allergies. After 14 days, the patient recovered from COVID-19 and 8 days later the oral lesions showed remission.

Case 6. Another case was that of a 35-year-old male, quarantined at home, who presented ageusia 6 days later and an oral ulcer on the tonsillar pillar, which caused mild odynophagia. Following the teleconsultation, the following information was obtained: this was the patient’s first episode of oral ulcers and he had no history of recurrent aphthous stomatitis or any other ulcerative disease of the mouth. The lesion was superficial and circular, covered by a fibrinopurulent membrane and surrounded by an erythematous halo. After 14 days of isolation the patient recovered completely.

Case 7. A 29-year-old male, isolated at home, presented ageusia on the sixth day of isolation. At teleconsultation, a superficial, painful ulcer, with a diameter of 1 cm and a whitish pseudomembrane, surrounded by an erythematous halo, was detected on the ventral portion of the tongue. After 6 days the patient recovered.

Case 8. Another 28-year-old male was placed in isolation at home; after 2 days of isolation, ageusia developed and, after 8 days, aphthous-like ulcers in the upper and lower labial mucosae were observed. After 2 days, another ulcer was observed on the right side of the tongue. The recommended treatment was 0.12% non-alcoholic chlorhexidine mouthwash. The oral lesions healed completely after 9 days.

The authors’ opinion was that the oral manifestations were directly associated with COVID-19 infection and/or the severely compromised state of the patients.

Cebeci Kahraman and Caskurlu [56] described a case of a 51-year-old male patient, treated with clarithromycin 500 mg b.i.d., who reported worsened sore throat symptoms 10 days after the onset. The oral examination revealed a largely erythematous surface in the oropharynx, a few petechiae on the hard palate, and numerous pustular enanthema, 1–3 mm in diameter, near the soft palate border, more prominent on the left side. The lesions resolved after a few days of antibiotherapy. The authors suggested that oral mucosa may be involved in COVID-19 symptoms.

Chaux-Bodard et al. [48] described the case of a 45-year-old female patient who presented an irregular asymptomatic ulcer on the tongue dorsum following a painful inflammation of a tongue papilla, which evolved in an erythematous macula. After 10 days, the ulcer showed complete remission. Three days after the debut of the oral lesion, a painful erythematous plane lesion also appeared on the big toe, which became asymptomatic after 2 days. The authors suggested that the macular erythematous lesion could be explained by vasculitis, an inflammatory reaction to COVID-19. Thus, the irregular oral ulcer could be considered as an inaugural symptom of COVID-19.

Ciccarese et al. [57] described the case of a previously healthy 19-year-old female, without comorbidities, who had started taking oral cefixime 3 days prior to admission. She reported asymptomatic cutaneous and oropharyngeal lesions that started 2 days before admission. Upon examination, the following lesions were detected: erythematous macules, papules and petechiae on the lower extremities, erosions, ulcerations, and blood crusts on the inner surface of the lips and palatal and gingival petechiae. The oral lesions were painless, heterogeneous in morphology, and associated with severe thrombocytopenia. Intravenous immune globulins and methylprednisolone were administered for 5 days, while antibiotic therapy was stopped. On day 10, the skin and mucosal lesions disappeared. The authors’ opinion was that the severe thrombocytopenia was probably of great importance in triggering the cutaneous and mucosal petechiae, while the oral erosions probable cause was direct viral vascular and mucosal damage.

Corchuelo and Ulloa [58] reported a case of a 40-year-old female, diagnosed positive for COVID-19 three weeks before the dental teleconsultation and treated with azithromycin. She presented painless reddish plaques on the lower lip and dark brown pigmentation of the gingiva. A whitish area was detected on the tongue dorsum, apparently accompanied by bacterial plaque. A well-defined brown band was observed in the attached gingiva, which did not transgress the mucogingival junction and partially affected the interpapillary gingiva. A painful aphthous ulcerative lesion on the attached lower left gingiva at the level of the first premolar was also detected. Petechiae were present on the upper part of the face. As treatment, nystatin oral suspension was prescribed, as well as rinses with chlorhexidine gluconate 0.12% and more frequent brush changes. Another dental teleconsultation was performed after 20 days: the recovery of the lesions of the lips was observed, no aphthous ulcers were present, and the whitish color of the tongue was significantly reduced. The melanin pigmentation in the attached gingiva of the anterior teeth was explained by the proliferation of melanocytes in that part of the body, as an inflammatory process induced by SARS-CoV-2.

Cruz Tapia et al. [59] reported a series of four cases with different oral manifestations.

Case 1. A 41-year-old female in home isolation, who had tested positive for SARS-CoV-2 and was treated with acetaminophen and fexofenadine, described discomfort in the palate. The clinical examination revealed an erythematous, asymptomatic, 6-mm-diameter, soft-consistency, nonbleeding bulla on the hard palate, diagnosed as an angina bullosa hemorrhagic-like lesion. The authors’ opinion was that the lesions were probably associated with COVID-19 and self-control measures were recommended.

Case 2. A hospitalized 51-year-old female presented diffuse vascular-like purple macule, 12 mm in size, on the left palatal mucosa and papule plaque of 8 mm on the right palatal mucosa. Dexamethasone, azithromycin, and indomethacin were administrated. The lesions, non-bleeding and asymptomatic, were considered a vascular disorder, probably associated with COVID-19.

Case 3. A 55-year-old female, isolated at home and treated with acetaminophen, noticed an enlargement of the tongue. Clinical examination revealed an asymptomatic purple bulla, 8 mm in diameter and of soft consistency, on the right side of the tongue, diagnosed as an angina bullosa hemorrhagic-like lesion, probably associated with COVID-19. The lesion presented a complete remission after 5 days.

Case 4. A 42-year-old male, confirmed positive for SARS-CoV-2, described associated burning-mouth symptoms. The oral examination showed multiple and irregular reddish macules, of 3–4 mm in diameter and indurated consistency, on the hard palate, and a nonspecific mucositis was considered. The patient received acetaminophen for 5 days, and clorhexidine 0.12% mouthwash was recommended. As the oral lesions persisted, after 14 days, an incisional biopsy was performed, leading to the diagnosis of mucosal, nonspecific, localized vasculitis and thrombosis associated with COVID-19. Topical mometasone furoate 0.1% and clorhexidine 0.12% mouthwash were prescribed, and after 7 days of treatment complete remission was observed.

Diaz Rodriguez et al. [60] reported three cases of oral manifestations in patients with confirmed COVID-19.

Case 1. A 43-year-old female was quarantined for 56 days. In the last 2 weeks, she reported aphthous-like lesions, a burning sensation, and tongue depapillation. Rinses with a triamcinolone acetonide 0.05% solution, three times a day for a 10-day period, were prescribed. After treatment, lingual depapillation persisted, but the ulcers and burning sensation disappeared.

Case 2. A 53-year-old man, a few days after being discharged from hospital, described a burning-mouth sensation and unilateral commissural fissures. Complaints of dysgeusia were also recorded. Lesions were diagnosed as commissural cheilitis and treated with an ointment containing neomycin, nystatin, and triamcinolone acetonide three times a day. Between ointment applications, use of a gauze with chlorhexidine for local hygiene was also described. Commissural lesions disappeared completely after treatment but dysgeusia persisted.

Case 3. A hospitalized 78-year-old woman reported a very intense sensation of dry mouth. Dental consultation revealed lesions on the tongue, palate, and commissure, compatible with pseudomembranous candidiasis and angular cheilitis. Solutions and gels to improve salivary dryness and nystatin solution rinses four times a day for 15 days were prescribed. Angular cheilitis was treated using ointment containing neomycin, nystatin, and triamcinolone acetonide. After treatment, the pseudomembranous lesions and commissural fissures healed and salivary flow and dry mouth sensation improved.

The authors noted that the cases were related to a certain state of immunosuppression and that stress might have played an important role in the appearance of these oral conditions. The authors’ opinion was that a cause–effect relationship between COVID-19 and the oral manifestations could not be established.

Dominguez-Santos et al. [61] presented four cases of young COVID-19 patients who developed minor aphthae during the course of the disease. The patients developed a low number of aphthae (ranging from one to more than five), measuring less than 1 cm, with a creamy-white fibrin surface and an erythematous peripheral ring, mostly affecting the non-keratinized mucosa. Only one patient had a history of recurrent aphthous stomatitis. Tests to exclude secondary causes of aphthosis were performed and PCR testing for HSV was negative. The authors stated that a causal association of oral aphthous ulcers with COVID-19 infection could not be demonstrated.

Glavina et al. [62] presented the oral manifestations related to COVID-19 in a 40-year-old female with no comorbidities and a history of frequent eruptions of recurrent herpes labialis. Dysgeusia was described as the initial symptom, followed by pain and burning sensation in the oral cavity 7 days after the patient was confirmed positive for SARS-CoV-2. The telemedical consultation revealed recurrent HSV of the hard palate, a white, hairy tongue, and non-specific white lesions of the ventral side of the tongue. Systemic acyclovir therapy was administrated five times a day for 5 days and local therapy (antiseptic, nystatin, panthenol, local anesthetic) for 2 weeks, until complete recovery. The authors’ opinion was that the recurrent HSV lesions were not caused, but triggered, by SARS-CoV-2 infection, as recurrent oral HSV infection is stress-induced and indicates a compromised immune system.

Jimenez-Cauhe et al. [63] reported cases of three patients who returned to the emergency department because of skin rashes 6, 7, and 4 days after being discharged, respectively. The oral cavity examination revealed palatal macules and petechiae. Following treatment with systemic corticosteroids, the erythema multiforme-like eruption showed progressive resolution after 2–3 weeks. The authors suspected an infectious cause rather than a drug reaction but could not positively exclude the involvement of the various drugs administered.

Kitakawa et al. [64] described the case of a female patient, 20 years old, who tested positive for COVID-19 and was treated with azithromycin and dipyrone. She showed lesions in the median lower lip semimucosa and severe pruritus, with a clinical course of 14 days. These lesions were treated with nebacetin ointment for 2 days, showing a good resolution. After a photographic examination, a differential diagnosis of recurrent herpes was established. The authors’ opinion was that it could have been a coincidence; however, the patient did not show any episodes of herpes infection in her history.

Labe et al. [65] described the cases of two children in which the cutaneous manifestations were at the forefront of the clinical picture.

Case 1. A 6-year-old male was hospitalized for painful cheilitis, which developed a week before admission and was followed by a rash of the extremities, and conjunctivitis. Oral examination revealed severe erosive cheilitis with diffuse gingival erosions and thick haemorrhagic crusts. The HSV test was negative. The diagnosis of erythema multiforme was established. As the child’s condition improved, he was discharged after 2 weeks.

Case 2. A 3-year-old male, hospitalized, showed cheilitis, stomatitis, and glossitis, accompanied by skin manifestations. After the diagnosis of COVID-19-associated Kawasaki disease was established, an initial dose of intravenous gamma globulin was administrated. Kawasaki disease is a systemic vasculitis with unknown etiology, associated with either viruses or bacteria. The authors stated that this case strongly suggests that SARS-CoV-2 is a trigger for Kawasaki disease and supports previous studies focused on possible associations between HCoV and Kawasaki disease [66,67].

Martin Carreras-Presas et al. [68] presented three cases of oral manifestations associated with COVID-19. In all cases, video consultations were performed.

Case 1. A healthy 56-year-old male patient, suspected of having COVID-19, presented dysgeusia, pain in his palate, and sore throat. Lesions resembling recurrent herpetic stomatitis were detected; however, the patient denied any HSV history. The prescribed treatment was valaciclovir for 10 days, and topical antiseptics with chlorhexidine and hyaluronic acid. After 10 days, the oral lesions showed full recovery.

Case 2. A 58-year-old male patient, suspected of having COVID-19, also reported pain in his palate. Multiple small, yellowish ulcers with an erythematous halo were detected on his palate. The patient did not have any previous history of herpetic infection. After 1 week of using topical antiseptic mouthwash, the painful lesions healed completely.

Case 3. A 65-year-old female patient, hospitalized and treated with antibiotics, corticosteroids, and antiviral drugs, developed a rash after being discharged. She described pain in her tongue from the beginning of the disease but had not been given an intraoral examination. After one week of antifungal administration, blisters in the internal lip mucosa, as well as desquamative gingivitis, could be observed. Hyaluronic acid and chlorhexidine mouthwash were prescribed, as well as prednisolone. The oral lesions improved within 3 days. In the authors’ opinion, it can be assumed that SARS-CoV-2 can provoke exanthematic lesions similar to other viral processes usually diagnosed in the dental clinic.

Nuno-Gonzalez et al. [69] provided the results of a cross-sectional study that aimed to evaluate cutaneous findings in 666 COVID-19 patients with mild-to-moderate pneumonia and a mean age of 55.7 years. A total of 304 of them presented one or more mucocutaneous manifestations. Oral manifestations, such as transient lingual papillitis, glossitis with lateral indentations or patchy depapillation, aphthous stomatitis, and mucositis, were also found in 78 cases. A burning sensation was also reported, and dysgeusia was commonly associated. The authors stated that, due to the frequency of the oral lesions, a specific examination was in order to avoid contagion risk.

The case of a 35-year-old female patient, who attended the dental emergency department describing fever, halitosis, intense gingival pain, and bleeding, was described by Patel [70]. Bilateral submandibular lymphadenopathy, severe halitosis, generalized erythematous and edematous gingivae, and necrotic interdental papillae in both the maxillary and mandibular labial sextants were accompanied by unprovoked bleeding from the gingival sulcus. A clinical diagnosis of necrotizing gingivitis was made and treatment with metronidazole three times daily for 5 days and 0.12% chlorhexidine mouthwash twice daily for 10 days was prescribed. Five days later, complete resolution of oral symptoms and fever was observed. Despite the fact that the patient was not tested and only a suspicion of COVID-19 infection could be stated, the authors emphasized the role of bacterial co-infections in COVID-19 severity.

Putra et al. [71] described a case of a 29-year-old male who presented cutaneous manifestations and was treated with azithromycin, hydroxychloroquine, oseltamivir, vitamin C3, and vitamin D1. Stomatitis aphthous, noticed on day seven, with no other treatment besides typical hygiene oral care, showed complete remission on day ten. The diagnosis of hand, foot, and mouth disease was supported by the appearance of stomatitis aphthous.

Riad et al. [72,73] presented two series of cases involving oral manifestations in patients confirmed to be infected with COVID-19.

The first series [72] consisted of 17 confirmed COVID-19 patients with angular cheilitis. The patients had a mean age 39.94 and 12 of them were female. The authors’ opinion was that angular cheilitis in COVID-19 patients can be attributed to numerous local irritants, including hypersalivation.

The second case series [73] included 26 confirmed COVID-19 patients with painful tongue ulcers. Their average age was 36.81, there were 9 males and 17 females, and they were treated with oral paracetamol and chlorhexidine mouthwash. The ulcers disappeared after 1–2 weeks. The authors’ opinion was that tongue ulcers can be a direct manifestation of SARS-CoV-2 infection or a co-infection due to the immune dysregulation.

Sakaida et al. [74] presented a case of a 52-year-old woman with itchy erythematous lesions on her limbs after being treated for 3 days with antibiotics and a non-steroid anti-inflammatory drug for previous dental problems. After 2 days of treatment, erosions on her lips and buccal mucosa appeared. The skin lesions were clinically diagnosed as a drug eruption. Oral prednisolone was administrated to treat oral lesions, which gradually improved. Five days after decreasing the prednisolone, symptoms of SARS-CoV-2 appeared and the patient tested positive. The authors’ opinion was that the drug eruption during the latency period might have been related to a COVID-19-induced cytokine storm.

Soares et al. [75] reported a case of a 42-year-old male patient, confirmed positive for SARS-CoV-2, who developed reddish oral lesions and a painful ulceration in the buccal mucosa, associated with petechia-like skin and small vesicobullous lesions of unknown etiology. The multiple reddish macules of different sizes were scattered along the hard palate, tongue, and lips. A treatment with dexamethasone and dipyrone was established for 1 week, and after 3 weeks the lesions presented complete remission. The authors suggested that SARS-CoV-2 can cause oral lesions and therefore that all positive patients should have a full mouth check-up.

The general aspects of the abovementioned studies are summarized in Table 1.

According to the reviewed data, the most frequent types of oral lesions found in COVID-19 patients are: ulcers (42 cases, 25.92%), aphthous stomatitis/aphtae (29 cases, 17.90%), angular cheilitis/cheilitis (21 cases, 12.96%), glossitis/lingual papillitis (21 cases, 12.96%), petechiae (8 cases, 4.93%), macules (7 cases, 4.32%), erythematous and erosive lesions (6 cases, 3.70%), herpetic lesions (6 cases, 3.70%), candidiasis (4 cases, 2.46%), and bulla (2 cases, 1.23%).

## 4. The Association between Periodontal Disease and COVID-19

Periodontal disease, a severe inflammatory gum disease, mainly affects the supporting structures of the teeth, gingiva, and alveolar bone, and it is frequently associated with poor oral hygiene and age. As the human organism normally responds to bacterial infection through inflammation, this process can result in a “cytokine storm”, where proteins are released and associated with an exuberant inflammatory response that destroy tissues in other parts of the body [76].

The inflammatory products can enter the bloodstream through periodontal pockets and reach other organs, causing tissue damage [77]. Pro-inflammatory cytokines and oxidative stress, involved in the development of periodontal disease and other metabolic diseases, are highly elevated among COVID-19 patients [78]. Bacteria in the gums spread the IL-6 inflammatory protein. High levels of IL-6 in the body are a predictor of respiratory failure, with a 22 times higher risk for respiratory complications being reported, thus highlighting the importance of reducing the amount of oral bacteria and subsequent systemic inflammation [79].

On the other hand, the high prevalence of periodontal disease among patients experiencing metabolic diseases, such as obesity and diabetes, and cardiovascular diseases is well-documented. These types of comorbidities, which affect systemic health, are also known to increase the risk for severe COVID-19 [80,81,82]. The association between periodontal disease and severe COVID-19 could help identify risk groups and establish pertinent recommendations [83].

A study on 568 patients, showed a clear association between periodontitis and increased levels of biomarkers associated with severe COVID-19 disease, as well as complications including death, ICU admission, and the need for assisted ventilation [84].

The investigations of a possible link between the microbial oral flora and COVID-19 also revealed that there is a risk that oral secretions may be aspirated into the lungs and cause infection [85]. Oral bacteria, such as the periodontal pathogens Porphyromonas gingivalis, Fusobacterium nucleatum, and Prevotella intermedia, may accelerate viral infectious diseases such as COVID-19 and aggravate lung damage [50]. Cytokines such as interleukin 1 (IL1) and tumor necrosis factor (TNF), which are present in the saliva as a consequence of their bacterial activity, can easily reach the lungs [84].

Poor oral hygiene, a frequent consequence of low income or psychological troubles, can lead to COVID-19 aggravation due to the aspiration of periodontopathic bacteria, which induces the expression of ACE-2, a known receptor for SARS-CoV-2, and the production of inflammatory cytokines in the lower respiratory tract. Long-term hospitalization of patients with COVID-19 leads to reduced professional oral care. Poor oral hygiene, and limited access to dental care in patients with COVID-19, may increase the inter-bacterial exchanges between the oral cavity and the lungs and thus the risk of a much more severe respiratory infection [86,87,88].

**Figure 2:**
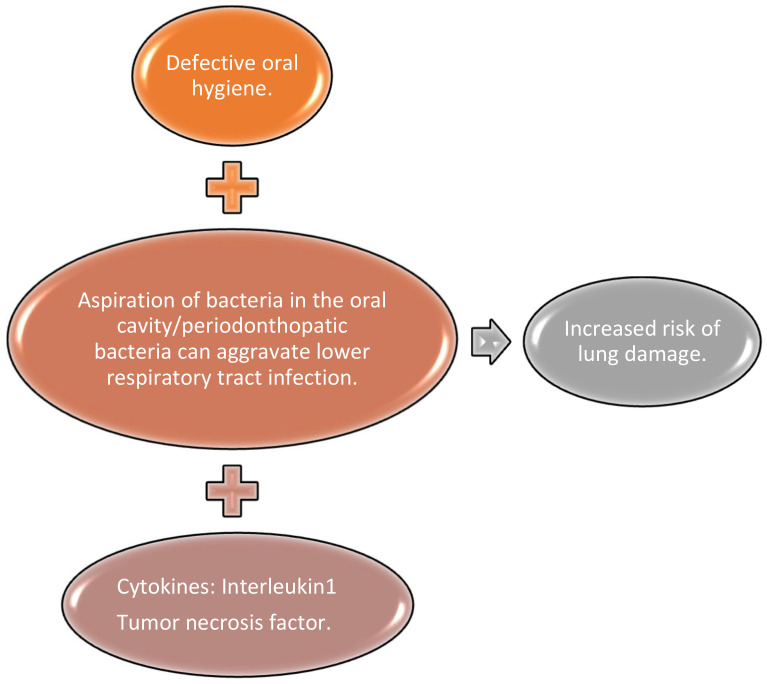
The link between poor oral hygiene, bacteria present in the oral cavity, and increased risk of lung damage.

The degree of periodontal inflammation may help to determine the severity of COVID-19 infection. Routine dental and periodontal treatment may also help decrease the symptoms of COVID-19 [50,85]. The link between poor oral hygiene, bacteria present in the oral cavity, and increased risk of lung damage is presented in Figure 2.

## 5. Temporomandibular Disorders Associated with COVID-19 Pandemic

Among the most common symptoms of temporomandibular disorders (TMDs) are soreness in the jaw joint area and jaw muscles and clicking or crunching noises when opening or closing the mouth or when the patient chews, yawns, or even speaks. TMD may be linked with headaches, neck pain, and discomfort in the temple or teeth. TMD reflects the dysfunction of the masticatory system, one of its major causes being stress and psychosocial impairment [89].

Pandemics are stressful, like most public health emergencies. The literature presents aspects of psychological reactions related to epidemics and pandemics, which depend on individual vulnerability, intolerance to uncertainty, perceived vulnerability to disease, and anxiety [90]. The anxiety, depression, and stress people experience during the COVID-19 pandemic may lead to TMD [91].

Uncertainties about the origin and nature of the virus and about governments’ abilities to prevent its spread, lack of confidence in the medical system and its ability to cope with new cases, fear of infection, misinformation, and feelings of loneliness and anger in quarantined people due to lack of socialization play important roles in the development and maintenance of TMD [92].

These psychosocial factors, often associated with sympathetic activity and additional release of adrenocortical steroids, may lead to muscle vasoconstriction and increased peripheral vascular resistance. Autonomic insufficiency can increase the sympathetic impulse and the feeling of hyper-excitement that creates and perpetuates sleep disorders, accompanied by sensations such as heat and cold, palpitations, tachycardia, nausea, abdominal pain, diarrhea, and constipation [93].

Reports have noted an increased number of people experiencing teeth grinding and oral pain during the COVID-19 pandemic as a consequence of increased stress due to health worries, the loss of work, and lockdown or separation from family members [94,95]. On the other hand, stress, anxiety, and depression due to COVID-19 lead to increased orofacial pain, TMD, and bruxism symptoms [96,97].

According to another recent study, people with chronic TMD are more susceptible to COVID-19 distress, resulting in deterioration of their psychological status, and increased chronic facial pain severity, supporting the hypothesis that stress acts as an amplifier of central sensitization, anxiety, depression, chronic pain, and pain-related disability in TMD cases [98].

Two concomitant studies aimed to evaluate the effect of lockdown on TMD and bruxism symptoms among 700 subjects from Israel and 1092 from Poland, respectively, by using online questionnaires. The results showed significant altered psychoemotional status, leading to aggravated bruxism and TMD symptoms, accompanied by increased orofacial pain [97].

Even after the lockdown period ended, patients with high risk for severe COVID-19 limited their dental appointments to emergencies only, which was not the case for TMD and bruxism. As they were neglected, these conditions got worse [99].

Medical staff, including dental practitioners, have also been reported to experience moderate to severe levels of anxiety because of possible COVID-19 repercussions [90,94,100].

A study carried out on 641 dental surgeons found TMD in 24.3% of the participants, sleep bruxism in 58%, and awake bruxism in 53.8% [101]. The incidence of TMD reported by a study carried out on 699 dental university students during the COVID-19 pandemic was of 77.5%, accompanied by impaired sleep quality, depression, anxiety, and stress [102]. Another study, based on 113 questionnaires filled out by dental students, also reported that the social isolation and stress due to the COVID-19 pandemic had led to increase symptoms of TMD, anxiety, and depression [103].

## 6. Dental Medicine during the COVID-19 Pandemic

Health systems around the world were subjected to a great challenge due to the rapid spread of SARS-CoV-2 and the related COVID-19 pneumonia until the vaccine became available. The public health measures during the pandemic forced patients with and without SARS-CoV-2 to remain isolated in order to prevent the spread. The majority of the patients were unable to attend dental services, postponed the appointments, and even neglected their oral hygiene, which can lead to complications [58].

At the beginning of the pandemic, dentistry, as well as oral and maxillofacial surgery and dental radiology, were included among the groups with the highest risk of infection due to inevitable close contact with SARS-CoV-2 asymptomatic and symptomatic patients [104,105].

Dental staff may develop an increased risk of infection due to the proximity of patients, who cannot wear masks during treatment and keep their mouth open [106]. The high risk is also caused by instruments and equipment that generate aerosols that contain oral and respiratory fluids, such as high-speed and ultrasonic scaling devices, both of which use a water-coolant [86,106,107,108]. Dental radiology, which does not allow the use of a rubber dam, is equally risky, as the patient may cough or gag if the image receptor is placed deep inside the mouth [109].

The high viral load in the nasal cavity in infected patients, even if asymptomatic, puts dentists and maxillofacial surgeons at even higher risk for SARS-CoV-2 infection because of the close contact. Treating patients in these pandemic times has to be undertaken with maximum precautions in order to minimize the infection transmission [110,111,112].

For a determined period of time at the beginning of the pandemic, dental practices were closed and only dental emergencies that could not be postponed [95] or facial trauma surgery were performed [112,113].

The most common types of facial trauma, generally caused by road or sports accidents, were reduced during this period because of the imposed restrictions, but trauma caused by domestic violence or falls still occurred. Oral and maxillofacial surgeons, head and neck surgeons, and plastic surgeons managed both facial trauma and patient triage by performing COVID-19 buffer tests and helping intubate COVID-19-positive patients with facial trauma [110].

During the peak of the pandemic, when personal contact was avoided as much as possible, telemedicine proved to be a useful tool in dental diagnosis [60,112].

Later on, specific protocols were implemented, combining sanitizing procedures with the wearing of disposable personal protective equipment (PPE). Patient screening by telephone, before scheduling a dental appointment, has been considered necessary to prevent spreading the virus inside the dental office [114,115].

At their arrival, patients must wear a surgical mask and must be unaccompanied (when possible). Patients are requested to leave any personal belongings in certain spaces and sanitize their hands, and they are provided with PPE.

The dental staff have to be equipped with disposable personal protective equipment: gloves, filtering facepiece particle 2 (FFP2) respirator, visor, protective gown, and shoe covers [116,117]. 

In order to reduce the presence of the virus in the saliva, rinsing with a mouthwash for at least 30 s prior to starting the dental treatment has been advised [118].

During clinical procedures, the use of a rubber dam is strongly recommended to limit the spread of aerosols and potentially infected biological material. The procedures which might cause coughing or gagging must be avoided; for example, the use of an intra-oral scanner instead of a conventional dental impression is preferred [109].

The type of aspirating system used seems to affect the prevalence of SARS-CoV-2 infection across dental offices. Using aspirating systems equipped with HEPA filters, capable of evacuating and dissipating aerosols into specialized areas, is strongly recommended [119].

At the end of the dental appointment, all disposable PPE must be properly discarded [120].

Accurate sanitizing of hands and of all surfaces is equally important, as well as proper ventilation for patients [120,121,122].

Radiography practices should be kept as simple as possible and minimize staff-to-patient contact. Intraoral radiographs should be avoided as much as possible during the COVID-19 pandemic. Extraoral bitewings represent an alternative for sectional panoramic radiographs and intraoral bitewings. Extraoral bitewings, which involve a radiation dose lower than or comparable to intraoral radiographs while providing a greater field of view, could be further considered, especially in cases of children and adults with difficulties in tolerating intraoral radiographs [123].

## 7. Conclusions and Future Perspectives

Oral manifestations related to COVID-19, including fungal infections, recurrent HSV, oral ulcerations, drug-related eruptions, dysgeusia, xerostomia or decreased salivary flow, and gingivitis, may be a result of the impaired immune system and/or susceptible oral mucosa [124].

Although, it is difficult to state which of the various oral lesions associated with COVID-19 are the most prevalent, it seems that a higher frequency can be found in older, hospitalized patients with severe infection [125].

A number of factors, such as immune impairment, co-morbidities, poor oral hygiene, adverse drug reactions, stress, secondary hyper-inflammatory responses, and iatrogenic trauma following intubation, may be involved [126].

The hypothesis that the oral manifestations are secondary lesions resulting from the deterioration of systemic health or treatments for COVID-19 is most probably correct. The pharmacological agents against COVID-19 are related to several adverse reactions, including oral lesions [127].

The authors of one study stated that “The oral mucosal examination has been neglected during the pandemic on reasonable grounds” [56]. A routine intraoral examination should always be performed on patients with suspected or confirmed SARS-CoV-2 infection, as it can represent a sign of potentially life-threatening conditions [57,70].

It has also been stated that “Whether the currently emerging new viral variants will have an impact on the oral manifestations is unknown” [125]. It is our belief that extensive knowledge about all possible manifestations in cases of COVID-19, including oral lesions, is of great importance in the present uncertain context. The fourth wave of COVID-19 and the alarming spreading of the Delta strain, which is highly contagious and potentially severe, keep the subject actual; skin manifestations are increasingly frequent. It can be assumed, based on the correlation between skin and oral manifestations, that new outcomes regarding this subject will emerge.

Dentists are not only implicated in providing specialty assistance in times of pandemics but also in fighting against them. A special note that seems worth mentioning is that, during the anti-COVID-19 vaccination campaign in Romania, marathon vaccinations were carried out during weekends in an effort to encourage attendance and limit the spread of the pandemic. Dentists took part as volunteers, together with fellow doctors and students, and 6722 people were vaccinated in the authors’ hometown during the first organization of this marathon series.

## Figures and Tables

**Table 1:** Main aspects of the included studies.

Study	Number of Cases	Patient Data	Oral Lesion	Localization
Amorim dos Santos et al. [51]	1	M, 67, confirmed	White plaque Yellowish pinpoint ulcers Geographical tongue	Tongue dorsum
Ansari et al. [53]	2	1. M, 75, confirmed 2. F, 56, confirmed	Several painful ulcers, with irregular margins and varying sizes against red and nonhemorrhagic backgrounds	1. Hard palate 2. Anterior region of the tongue
Bezerra et al. [54]	1	M, 33, suspicion	Painful mouth ulceration Two crateriform ulcers with a necrotic background and no erythematous halo	The floor of the mouth Left retromolar region and lip mucosa
Brandao et al. [55]	8	1. M, 81, confirmed 2. F, 71, confirmed 3. F, 83, confirmed 4. M, 72, confirmed 5. F, 32, confirmed 6. M, 35, confirmed 7. M, 29, confirmed 8. M, 28, confirmed	1. Multiple painful, shallow aphthous-like ulcers covered with mucopurulent membrane HSV-1 identified 2. Small hemorrhagic ulcerations Areas of superficial necrosis HSV-1 identified 3. Ulcer of 1.5 × 1.5 cm^2^ Discrete area affected by petechiae and a superficial necrotic area 4. Small hemorrhagic ulcerations Painful necrotic ulceration HSV-1 identified 5. Multiple ulcers, superficial and circular lesions with a whitish center and surrounded by an erythematous halo 6. Superficial, circular ulcer, covered by a fibrinopurulent membrane and surrounded by an erythematous halo of 0.5 cm Mild odynophagia. 7. Superficial, painful ulcer, with a diameter of 1 cm and a whitish pseudomembrane surrounded by an erythematous halo 8. Aphthous-like ulcers	1. The mucosa of the upper and lower lips Anterior tongue dorsum 2. Upper and lower lip Anterior tongue dorsum 3. Right lateral edge of the tongue Anterior hard palate 4. Upper and lower lip Lower right lip 5. Tip and lateral edges of the tongue 6. Tonsillar pillar 7. Ventral portion of the tongue 8. Upper and lower labial mucosae, right side of the tongue
Cebeci Kahraman and Caskurlu [56]	1	M, 51, confirmed	Large erythematous surface A few petechiae Numerous pustular enanthemata	Oropharynx Hard palate midline Left side of soft palate border
Chaux-Bodard et al. [48]	1	F, 45, confirmed	Painful inflammation of tongue papilla, followed by an erythematous macula and an asymptomatic irregular ulcer	Tongue dorsum
Ciccarese et al. [57]	1	F, 19, confirmed	Erosions, ulcerations, and blood crust Petechiae	The inner surface of the lips Palate and gingiva
Corchuelo and Ulloa [58]	1	F, 40, confirmed	Reddish plaques Dark brown pigmentation Aphtous-like ulcer White area, probably candida	Lower lip Gingiva Attached lower left gingiva Tongue dorsum
Cruz Tapia et al. [59]	4	1. F, 41, confirmed 2. F, 42, confirmed 3. F, 55, confirmed 4. M, 41, confirmed	1. Bulla 2. Macule 3. Bulla 4. Small macule	1. Hard palate 2. Hard palate (left side) 3. Tongue 4. Hard palate
Diaz Rodriguez et al. [60]	3	1. F, 43, confirmed 2. M, 53, confirmed 3. F, 78, confirmed	1. Aphthous-like lesions, burning sensation, and tongue depapillation 2. Burning-mouth sensation and unilateral angular cheilitis 3. Pseudomembranous candidiasis and angular cheilitis	1. N/A 2. Lips 3. Tongue, palate
Dominguez-Santos et al. [61]	4	1. F, 43, confirmed 2. M, 33, confirmed 3. M, 37, confirmed 4. M, 19, confirmed	1. Single ulcer, with peripheral erythematous rim 2. Single aphtous ulcer 3. Seven aphtae 4. Four clustered aphtae	1. Right buccal mucosa 2. Superior mucogingival junction 3. Ventral right side of the tongue 4. Right side of the inferior labial mucosa
Glavina et al. [62]	1	F, 40, confirmed	Pain and burning in the oral cavity Recurent HSV White, hairy tongue	Hard palate Tongue
Jimenez Cauhe et al. [63]	3	F, 77, confirmed F, 58, confirmed F, 69, confirmed	Macules and petechiae	Palate
Kitakawa et al. [64]	1	F, 20, confirmed	Herpetic lesions, pruritus	Median lower lip semimucosa
Labe et al. [65]	2	1. M, 6, confirmed 2. M, 3, suspicion	1. Painful cheilitis 2. Cheilitis, stomatitis, glossitis	N/A N/A
Martin Carreras-Presas et al. [68]	3	1. M, 56, suspicion 2. M, 58, suspicion 3. F, 65, confimed	1. Painful lesions resembling a herpetic recurrent stomatitis 2. Multiple small, painful yellowish ulcers with erythematous halo 3. Pain Blisters Desquamative gingivitis	1. Palate 2. Palate 3. Tongue Internal lip mucosa Gingiva
Nuno-Gonzalez et al. [69]	78	Adults, average age of 55.7 years, confirmed/suspicion	Lingual papillitis, glossitis, aphthous stomatitis, mucositis, and burning sensation	N/A
Patel et al. [70]	1	F, 35, suspicion	Erithematous and edematous gingiva and necrotic interdental papillae	Gingiva and interdental papillae
Putra et al. [71]	1	M, 29, confirmed	Aphthous stomatitis	N/A
Riad et al. [72]	17	12 F, 5 M; average age of 39.94, confirmed	Angular cheilitis	Lips
Riad et al. [73]	26	9 M, 17 F; average age of 36.81, confirmed	Tongue ulcers	Tongue
Sakaida et al. [74]	1	F, 52, confirmed	Erythematous and erosive lesions	Lips and oral mucosa
Soares et al. [75]	1	M, 42, confirmed	Painful ulceration Multiple reddish macules of different sizes	Buccal mucosa Hard palate, tongue, and lips
